# Oral Manifestations Associated with COVID-19 Infection: A Cross-Sectional Study of Recovered Iraqi Patients

**DOI:** 10.1155/2023/4288182

**Published:** 2023-02-17

**Authors:** Mohanad Jameel Najm Al-Magsoosi, Oras Kadhim Baqer Al-Asadi, Nibrass Talib Al-Quraine, Suha Mohammad Sami, Julfikar Haider

**Affiliations:** ^1^Department of Oral Diagnosis, College of Dentistry, University of Basrah, Basrah, Iraq; ^2^Department of Dentistry, Al-Manara College for Medical Sciences, Amarah, Iraq; ^3^Department of Conservative Dentistry, College of Dentistry, University of Kufa, Najaf, Iraq; ^4^Department of Maxillofacial Surgery, College of Dentistry, University of Kufa, Najaf, Iraq; ^5^Department of Engineering, Manchester Metropolitan University, Manchester, UK

## Abstract

**Aims:**

The aim of this study was to determine prevalence of oral manifestations related to COVID-19 infection among a sample of recovered patients in the Basrah province of Iraq. *Methodology*. This cross-sectional study included a total of 574 individuals from Basrah city, Iraq (196 males and 378 females), who had been previously infected with COVID-19. A questionnaire was developed and used to record the demographic data, medical history, severity of respiratory infection followed by hospitalization along with oral signs and symptoms that occurred during the COVID-19 infection and their persistence after recovery.

**Results:**

Oral manifestations were reported in 88.3% of the studied sample. The most common oral manifestation was ageusia (66.8%), followed by dry mouth (59%), gustatory changes (46%), dysphagia (40.5%), burning sensation (20.8%), oral ulceration (14.5%), and gingival bleeding (3.3%). The findings suggested that ageusia was the only symptom that persisted following recovery from the COVID-19 infection. The results showed a significant statistical correlation between the incidence of oral manifestations and the severity of COVID-19 infection followed by hospitalization. A significant correlation was also found between the age groups and COVID-19 oral manifestations, whereas no significant statistical relationship was observed between gender, smoking, and systemic diseases.

**Conclusions:**

COVID-19 infection has considerable impacts on the oral cavity and salivary glands and after recovery from the infection, some patients continue to complain of ageusia for several months. There is a positive correlation between the incidence of oral signs and symptoms associated with COVID-19 infection and the severity of the infection.

## 1. Introduction

It has been more than two years since the recording of the first case of the novel coronavirus disease (COVID-19) in Wuhan City, China, and the declaration by the World Health Organization (WHO) of pandemic disease [1] but we could not identify the Wuhan City case as the truly first case all over the world. Since that time, approximately 643 million infection cases worldwide with more than 6 million deaths had been reported [2]. Fever, sore throat, severe headache, loss of smell and taste, shortness of breathing, dry cough, and tiredness are considered as the most common symptoms associated with the COVID-19 infection [3]. This viral disease is transmitted from human to human through respiratory droplets [4]. Previous studies have established that the SARS-CoV-2 virus invades human cells through an interaction with the host angiotensin-converting enzyme 2 receptors (ACE2) [5]. These receptors are expressed by a variety of human tissues such as lung, gastrointestinal tract, liver, kidney, skin, and the oral cavity [1, 3]. Furthermore, the oral cavity has a crucial role in the dissemination of SARS-CoV-2 virus since it serves as a gateway to the internal environment of the human body. It has been suggested that oral manifestations associated with COVID-19 may be either related to the direct effect of the SARS-CoV-2 on the tissues of the oral cavity or an indirect effect due to an impairment of the immune system by the virus [6, 7]. Oral manifestations related to COVID-19 infection have important negative impacts on nutrition, quality of life, and the psychological status of patients. In addition, they have an important role in the clinical diagnosis of disease [8].

Therefore, the aim of this cross-sectional study is to examine the types and prevalence of oral manifestations associated with COVID-19 and evaluates their persistence. In addition, any correlation between the incidence of these oral manifestations and several factors such as: gender, age, systemic disease, smoking, and severity of the infection have been investigated.

## 2. Methodology

### 2.1. Survey Procedure

This cross-sectional study was conducted on patients attending dental clinics of the College of Dentistry, University of Basrah, between October 2021 and April 2022. All the participants had been previously infected with COVID-19 and their infection was confirmed by a positive reverse transcription-polymerase chain reaction (RT-PCR) test.

Ethical approval was obtained by the Research Ethics Committee, College of Dentistry, Basrah University (BDC/07/09/2021), and the study was performed in accordance with Helsinki Declaration. All the participants were volunteers and had completed and signed a consent form.

The study sample consisted of a total 574 patients from Basrah province (196 males and 378 females), with an age range between 18 and 78 years.

A questionnaire was filled out to collect information from each patient. The patients were asked about their sex, age, occupation, address, smoking habits, the presence of any systemic diseases such as cardiovascular, respiratory, diabetes, cancer, kidney, and autoimmune disease and whether the respiratory infection was severe and required hospitalization. Details of any oral manifestations that occurred during the COVID-19 infection were recorded along with whether these persisted following the recovery.

### 2.2. Statistical Analysis

Data were statistically analyzed using Microsoft Excel software. The frequency and percentage were used to calculate the numerical data, whereas the Chi-square test was used to test significant statistical correlations. A *p* value of <0.05 was considered significant.

## 3. Results

The results showed that 507 patients (88.3% of the total number of COVID-19 patients studies), had at least one manifestation related to the oral cavity and salivary glands. Whereas only 67 (11.7%) did not report any sign or symptom associated with oral cavity.

**Figure 1:**
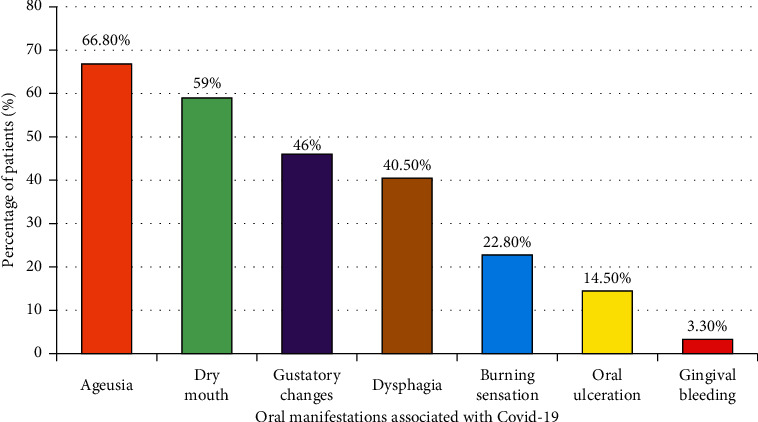
Percentage of oral manifestations.

The most common oral manifestation was ageusia (66.8%), followed by dry mouth (59%), gustatory changes (46%), dysphagia (40.5%), burning sensation (20.8%), and oral ulceration (14.5%). The least prevalent symptom was gingival bleeding (3.3%), and the percentage of oral manifestations is shown in Figure 1.

**Figure 2:**
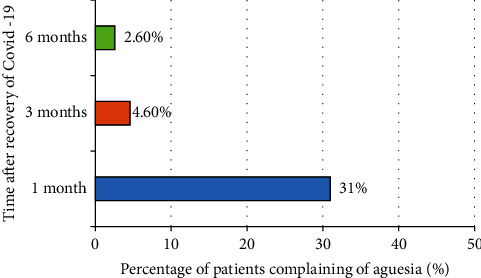
Percentage and duration of patients' complaints from ageusia following infection.

The findings showed that ageusia was the only symptom that persisted following recovery in 31% of the total patients who complained from ageusia for approximately one month, 4.6% for 3 months and 2.6% after 6 months (Figure 2).

Regarding the gender of the patients, 172 (87.7%) males and 335 (88.6%) females had at least one oral sign and symptom associated with the COVID-19 infection. There was no statistically significant difference in the prevalence between the males and females (*p*=0.7) as shown in Table 1.

With respect to the age groups, it was noticed that the most affected age group was 30–39 years (93%), followed by 40–49 years (92.2%), and 18–29 years (90%), respectively. While the least affected age group was 50–59 years. There is a statistically significant difference between the age groups (*p*=0.01, Table 2).

With regards to the presence of systemic diseases, the results showed that 128 patients in the study sample had systemic diseases and 112 (87.5%) of them complained of at least one oral manifestation during their COVID-19 infection. On the other hand, 446 participants had no systemic diseases, and 395 (88.6%) of them showed oral signs or symptoms during the period of infection. No statistically significant difference was detected between the patients with systemic diseases and healthy patients (*p*=0.7, Table 3).

Among 67 smokers in the study sample, 61 (91.1%) of them had at least one oral sign and symptom. Whereas the number of nonsmoker patients who were affected by oral manifestations was 446 forming 87.9% of the total number of nonsmoker patients. There was no statistically significant difference recorded between smoking patients and nonsmokers (*p*=0.4, Table 4).

Finally, the findings showed that 69 patients in the study sample was hospitalized during their COVID-19 infection due to the severe respiratory infection with 67 (97.1%) of them complaining of oral manifestations. 505 patients did not require admission to hospital and 440 (87.1%) of them had oral manifestations, a significant statistical difference between both groups (*p* = 0.01, Table 5).

## 4. Discussion

COVID-19 infection has effects on multiple organs and sites including oral cavity [1]. Although many studies have been published to investigate its general impacts, oral manifestations are still underestimated and unclarified [9]. Therefore, the rationale of the current study was to investigate the most common oral manifestations that are accompanied by the disease, their prevalence as well as the presence of any correlations with demographic factors and the severity of disease in an Iraqi cohort.

The results of the study showed that 66.8% of the examined patients had ageusia (complete loss of taste sensation) which is higher than that found by Ganesan et al. (51.2%) [8] and Natto et al. (43.4%) [10]. Moreover, in this research, 46% of the patients reported gustatory impairment, which is in accordance with that found by Amorim dos santos et al. (45%) [1] and comparable to that by Ganesan et al. [8] which was 51.2% and higher than that reported by El Kady et al. (34.5%) [11]. Two hypotheses could explain the high incidence of ageusia and gustatory changes during COVID-19. First, high expression of ACE2 receptors in epithelial cells in taste buds of the tongue that binds SARS-CoV-2 virus results in inactivation of these receptors and as a consequence, the taste perception is alter [12–14]. The second hypothesis suggests that taste loss might be attributed to side effects of medications used in the treatment of COVID-19 infection [15, 16]. However, the later hypothesis is considered questionable and remains unsupported due to the occurrence of ageusia and gustatory changes in drug-free COVID-19 patients with mild and moderate infection [12].

Dry mouth (xerostomia) is the second most common oral finding found in 59% of the total study sample which is comparable to that found by Ameen et al. (56%) but higher than that revealed by El Kady et al. (39.7%) [11] and Ganesan et al. (28%) [8]. Recent literature attributed the incidence of xerostomia during COVID-19 infection to prolong antibiotic treatment and poor hydration as well as the presence of *Candida* infection [8].

The results of the current study showed dysphagia in 40.5% of the participants which is higher than that reported by El Kady et al. (22.4%) [11] and Muthyam et al. (16%) [17]. Muthyam et al. established that swallowing difficulties and dysphagia are neurological complications of COVID-19 infection resulting from damage to the swallowing neural circuits [17].

In this study, the prevalence of burning sensation is 22.8% which is similar to that found by El Kady et al. (22.4%) [11]. Several studies suggested that burning sensation is a nonspecific symptom that might occur during COVID-19 infection as a consequence of other conditions such as dry mouth, candidal infection, oral ulceration, and any secondary infection [1, 18].

Our findings showed that 14.5% of the studied sample complained of oral ulceration following COVID-19 infection which is comparable to that found by El Kady et al. (17.2%) [11] and higher than that found by Natto et al. (6.4%) [10]. Previous studies have considered oral ulceration to be a nonspecific symptom and indirectly related to COVID-19. This could be due to several reasons such as blister as a result of high fever, aphthous ulcers triggered by stress, and recurrent herpes type 1 infection due to a compromised immune system during COVID-19 infection [19–22].

The last oral manifestation investigated in this study was gingival bleeding which is found in 3.3% of the study sample, which is less than that reported by El Kady et al. (7%) [11]. Gingival bleeding could be explained by the fact that COVID-19, like other debilitating diseases leads to reduced measures of maintaining oral hygiene. This leads to the accumulation of dental biofilm which is associated with an increased inflammatory reaction and accompanied by clinical signs of gingivitis [23].

To the authors' best knowledge, no previous study has examined the persistence of oral signs and symptoms following recovery from the infection. Our results demonstrated that only ageusia continued in some patients for up to 6 months following the recovery. There is no specific explanation for this as the exact pathogenesis of loss of smell and taste in COVID-19 is unknown [24]. Furthermore, many studies established that loss of taste accompanied olfactory dysfunction and most individuals considered both symptoms as a single entity rather than two [25].

In this study, the relationships between the incidence of oral manifestations with gender, age, smoking, systemic diseases, and the severity of infection were evaluated. The results showed a significant statistical correlation between oral manifestations and age of patients as well as the severity of infection. These findings are in consistent with the study by Ganesan et al. [8] who found no correlation between oral manifestations and gender and smoking, whereas a significant correlation was found with severity of the infection. In addition, the findings are in accordance with that reported by El Kady et al. [11] who showed that there is no correlation between genders with oral manifestations.

It is obvious in the current study that the high frequency of the oral manifestations reported by hospitalized patients. This could be attributed to the fact that hospitalized patients had severe acute respiratory infection, and most of them were intubated and are likely to have an impaired immune response. Furthermore, those patients were debilitated and had a long hospital course. All these factors lead to compromised oral hygiene and a subsequent increased incidence of oral signs and symptoms [8].

## 5. Conclusions

It is evident that COVID-19 infection has significant effects on the oral cavity and salivary glands. These effects increased with severity of the infection and patient hospitalization. In addition, some patients continue to complain of loss of taste for several months after recovery from the infection. Therefore, it is recommended that the dentists should have the adequate knowledge of the patterns of presentation and management of common oral manifestations associated with COVID-19. Furthermore, it is also suggested that dentists should be part of the COVID-19 therapy team so as to improve the process of recovery and the quality of life for the affected patients.

## Figures and Tables

**Table 1:** Frequency, percentage, and the statistical difference of examined and affected males and females.

Oral manifestations	*Gender*						*p*=0.7 nonsignificant
	*Male 196*		*Female 378*		*Total 574*		
	Frequency	%	Frequency	%	Frequency	%	
Yes	172	87.7	335	88.6	507	88.3	
No	24	12.3	43	11.4	67	11.7	
Total	196	100	378	100	574	100	

**Table 2:** Frequency, percentage, and the statistical difference of occurrence of oral symptoms in COVID-19 patients according to the age groups.

Age groups	*Oral manifestations*						*p*=0.01 significant
	*Yes*		*No*		*Total*		
	Frequency	%	Frequency	%	Frequency	%	
18–29	198	90	22	10	220	38.3	
30–39	93	93	7	7	100	17.4	
40–49	106	92.2	9	7.8	115	20	
50–59	67	77.1	20	22.9	87	15.2	
>60	43	82.7	9	17.3	52	9.1	
Total	507	88.3	67	11.7	574	100	

**Table 3:** Frequency, percentage, and the statistical difference of occurrence of oral symptoms in COVID-19 patients according to their medical condition.

Oral manifestations	*Presence or absence of systemic diseases*						*p*=0.7 nonsignificant
	*No systemic diseases*		*Systemic diseases*		*Total*		
	Frequency	%	Frequency	%	Frequency	%	
Yes	395	88.6	112	87.5	507	88.3	
No	51	11.4	16	12.5	67	11.7	
Total	446	100	128	100	574	100	

**Table 4:** Frequency, percentage, and the statistical difference of occurrence of oral symptoms in COVID-19 patients according to the smoking status.

Oral manifestations	*Smoking habit*						*p*=0.4 nonsignificant
	*Nonsmoker*		*Smoker*		*Total*		
	Frequency	%	Frequency	%	Frequency	%	
Yes	446	87.9	61	91.1	507	88.3	
No	61	12.1	6	8.9	67	11.7	
Total	507	100	67	100	574	100	

**Table 5:** Frequency, percentage, and the statistical difference of occurrence of oral symptoms in COVID-19 patients according to hospitalization.

Oral manifestations	*Hospitalization history*						*p*=0.01 significant
	*Non Hospitalized*		*Hospitalized*		*Total*		
	Frequency	%	Frequency	%	Frequency	%	
Yes	440	87.1	67	97.1	507	88.3	
No	65	12.9	2	2.9	67	11.7	
Total	505	100	69	100	574	100	

## Data Availability

The data that support the findings of this study are available from the corresponding author upon reasonable request.
